# Familial multiple fetal cerebral arteriovenous malformations: a case report of maternal genetic susceptibility and fetal manifestation

**DOI:** 10.3389/fgene.2025.1570754

**Published:** 2026-01-05

**Authors:** Hao Wang, Yan Jiang, YanLin Chen, GongLi Chen

**Affiliations:** 1 Department of Obstetrics and Gynecology, Women and Children’s Hospital of Chongqing Medical University, Chongqing, China; 2 Department of Obstetrics and Gynecology, Chongqing Health Center for Women and Children, Chongqing, China; 3 Chongqing Municipal Health Commission Key Laboratory of Perinatal Medicine, Chongqing, China

**Keywords:** fetal multiple cerebral, multiple cerebral arteriovenous, whole exome sequencing, maternal genetic susceptibility, ENG gene variant

## Abstract

**Introduction:**

Cerebral arteriovenous malformations (AVMs) are rare vascular anomalies that can present significant clinical challenges, especially when occurring in multiple sites. Hereditary hemorrhagic telangiectasia (HHT), a genetic disorder may be caused by mutations in genes such as ENG (encoding endoglin) or ACVRL1 (ALK1), and less commonly SMAD4, is one condition that predisposes individuals to multiple AVMs. This case report investigates the role of maternal genetic susceptibility in the fetal manifestation of multiple cerebral AVMs.

**Case Presentation:**

A multiparous woman with embolized pulmonary AVMs underwent high-resolution fetal neurosonography at 29+4 weeks for maternal HHT risk and third-trimester screening flags (prominent venous structures). Targeted ultrasound revealed multiple cerebral AVMs with a dilated superior sagittal sinus. Trio-exome identified a heterozygous ENG variant in the mother, Sanger-confirmed and present in the fetus, consistent with direct genetic predisposition. Limb/ear minor anomalies (polydactyly, accessory auricle) prompted syndromic re-analysis (CM-AVM, JP-HHT overlap, PI3K-pathway, GLI3), which was negative for additional diagnostic variants.

**Conclusion:**

This case strengthens the link between familial ENG-mediated HHT and fetal cerebral AVMs, underscores the value of targeted third-trimester neurosonography in at-risk pregnancies, and clarifies variant-level evidence supporting causality.

## Introduction

Cerebral arteriovenous malformations (AVMs) are rare but significant vascular anomalies that can present a wide array of clinical challenges, especially when occurring in multiple sites. These malformations are characterized by abnormal connections between arteries and veins, bypassing the normal capillary network, and leading to increased risks of hemorrhage, ischemia, and neurological impairment (Ngam et al., 2019). While most AVMs are sporadic, familial cases are extremely rare, and their genetic underpinnings are not fully understood. Understanding the genetic factors contributing to familial AVMs is crucial for early diagnosis, management, and genetic counseling.

Hereditary hemorrhagic telangiectasia (HHT), which is caused by pathogenic variants in genes such as ENG (endoglin), ACVRL1 (ALK1), and less commonly SMAD4, is one such condition that predisposes individuals to multiple AVMs. HHT primarily affects vascular development and is associated with arteriovenous malformations in multiple organs, including the brain, lungs, and liver (Crist et al., 2018). The ENG gene mutation leads to defective vascular remodeling, contributing to abnormal arteriovenous connections (Anzell et al., 2024). This case report highlights the potential role of maternal genetic susceptibility in the fetal manifestation of multiple cerebral AVMs, with a focus on the pathogenic ENG variant identified in the mother.

The significance of this case lies in its exploration of the familial aspect of cerebral AVMs, raising awareness of the genetic risks that may predispose fetuses to these rare malformations. Furthermore, it underscores the importance of genetic testing in understanding the molecular basis of AVMs, which may provide valuable insights into the pathogenesis of fetal brain malformations and guide clinical decision-making for management and prevention in future pregnancies.

## Case report/case presentation

A 27-year-old multiparous woman (G2P1) was evaluated for fetal multiple cerebral AVMs. Her prior pregnancy resulted in a healthy 3-year-old son who is currently asymptomatic; the family opted for future clinical monitoring for the child rather than immediate genetic testing. She had a personal history of pulmonary angiography and embolization of pulmonary arteriovenous malformations. Her routine ultrasound examinations at 12 and 20 weeks of gestation during the current pregnancy were unremarkable. The detailed fetal cranial ultrasound at 29+4 weeks was performed specifically due to the maternal history of AVMs, prompting a targeted late-gestation assessment. She denied chronic conditions such as hypertension, diabetes, heart disease, thyroid disorders, asthma, or epilepsy. She also denied infections like hepatitis or tuberculosis, other surgeries, trauma, blood transfusions, and food or drug allergies. Her immunization history was unclear. Her husband was healthy with smoking and alcohol habits and no travel history. Her parents were healthy with no family history of genetic or infectious diseases.

In early pregnancy, there was no vaginal bleeding or exposure to teratogens or radiation. Fetal movements were felt around 18 weeks. Her blood type was AB Rh-positive. At 8+5 weeks, her aneuploidy screening was low risk (Trisomy 21 risk 1:3618). The mid-pregnancy screen was also low risk (1:38339). Her OGTT was 4.01-4.68-5.59 mmol/L, and routine urine analysis was normal.

**FIGURE 1:**
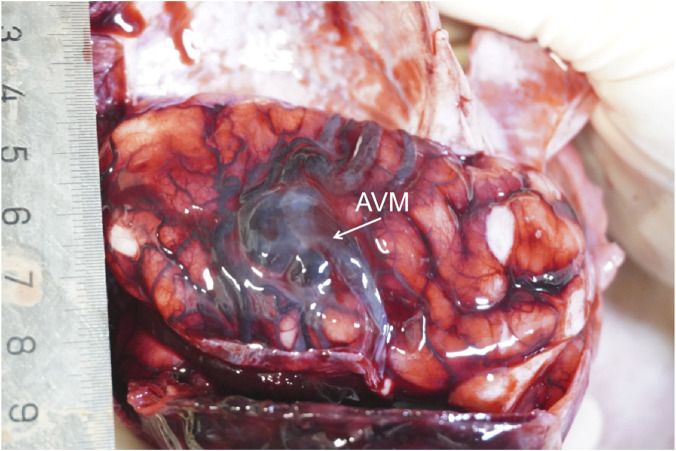
Postmortem gross examination of the fetal brain. The image shows markedly congested and dilated superficial cerebral vessels over the left hemisphere, with a visible tangle of vessels consistent with an arteriovenous malformation (AVM) nidus (arrow).

At 29+4 weeks, a fetal cranial ultrasound showed an oval-shaped echogenic skull, symmetric cerebral hemispheres, and a midline falx. The left lateral ventricle measured 0.77 cm, and the right was 1.21 cm. The cavum septum pellucidum was 0.56 cm wide. The thalami were symmetric. The cerebellar hemispheres and vermis were normal. The posterior fossa was not enlarged. Color Doppler revealed an abnormal circle of Willis, dilated and tortuous intracranial arteries (more prominent on the left), and a conglomerate of tortuous vessels measuring 2.0 × 1.7 cm in the left temporo-parietal region connecting to a dilated superior sagittal sinus (shown in Figure 1). The transverse and sigmoid sinuses were dilated bilaterally, as were the jugular and innominate veins.

**FIGURE 2:**
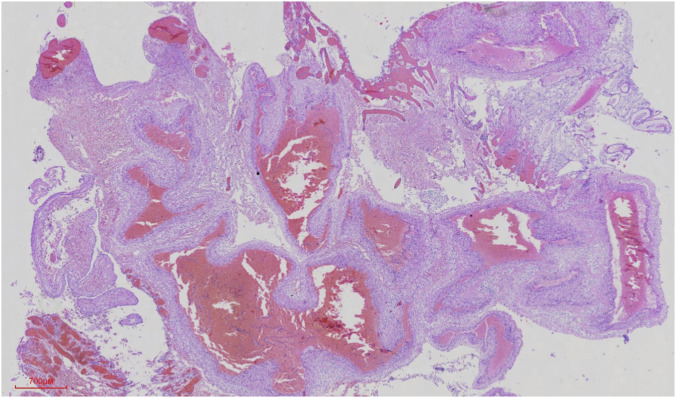
Histopathology of the fetal cerebral AVM (Hematoxylin and Eosin stain). The micrograph reveals a chaotic mixture of thick-walled arterial vessels and thin-walled, arterialized veins without an intervening capillary network, confirming the diagnosis of AVM.

At 29+6 weeks, termination of the pregnancy was performed by intraamniotic digoxin injection due to the poor prognosis. Two days later, the patient vaginally delivered a 1500g stillborn female with a 40 cm length and 50 cm cord without nuchal cord or torsion. The amniotic fluid was clear. The placenta delivered spontaneously. The infant had polydactyly of the left thumb and a right accessory auricle. A detailed postmortem examination was conducted. Gross examination of the brain revealed congested and markedly dilated superficial cerebral vessels, particularly over the left hemisphere. Upon sectioning, a large, complex tangle of abnormal vessels, consistent with an AVM nidus, was identified in the left temporo-parietal region, measuring approximately 2.1 × 1.8 cm. The malformation was associated with prominent, arterialized draining veins leading to a dilated superior sagittal sinus. Histological examination of the cerebral vessels confirmed the diagnosis, revealing a nidus composed of a chaotic mixture of thick-walled arterial vessels and thin-walled, arterialized veins without an intervening capillary network (Figure 2). The surrounding brain parenchyma showed evidence of gliosis and focal ischemic changes, likely secondary to vascular steal phenomenon. After surgery, sections and stains of the fetal cerebral vessels showed clear evidence of vascular malformation (shown in Figure 2).

Whole exome sequencing (WES) of the mother’s blood sample revealed a heterozygous likely pathogenic variant in the ENG gene [NM_001114753.3: c.1088G>A, p.(Cys363Tyr)] (shown in Table 1). This variant was subsequently confirmed by Sanger sequencing. Furthermore, genetic analysis of a fetal tissue sample obtained post-delivery confirmed the fetus was also a carrier of the same heterozygous ENG variant, establishing a direct genetic basis for the fetal phenotype. Pathogenic variants cause hereditary hemorrhagic telangiectasia (HHT), an autosomal dominant disorder characterized by multiple arteriovenous malformations that can lead to hemorrhage and vascular complications (Paquet et al., 2001).

**TABLE 1:** Details of the likely pathogenic variant identified in the ENG gene. The table presents the single heterozygous variant found in both the mother and the fetus, inherited in an autosomal dominant pattern. The variant’s classification was upgraded from VUS to “Likely Pathogenic” based on the strong co-segregation with the clinical phenotype in the family.

Gene	OMIM	Inheritance	HG19 position (Chr:Position)	Transcription	Nucleotide and amino acid changes	Zygosity	Source
ENG	131195	AD	chr9:130586629	NM 001114753.3 (Exon 8)	c.1088G>A (p.Cys363Tyr)	Heterozygous	Maternal

## Discussion

Most AVMs are sporadic and can occur anywhere in the brain or spinal cord. These arteriovenous shunting lesions have direct arterial flow into the nidus drained by multiple venous pathways, resulting in the characteristic “early venous drainage” seen on angiography. While the precise etiology is unknown, AVMs are thought to be embryologically acquired with significant anatomic heterogeneity between patients. Multiple AVMs are relatively rare, and their genetic basis remains to be fully elucidated with few reported familial clustering cases. AVMs have been associated with conditions like Osler-Weber-Rendu, Wyburn-Mason, von Hippel-Lindau, and Sturge-Weber syndromes. Despite proposed formation mechanisms, their exact cause often remains idiopathic due to lesion diversity and lack of defined genetic underpinnings (Osbun et al., 2017).

The pathophysiologic hallmarks of AVMs include a nidus formed by a convoluted venous tangle. These draining veins typically arise at the nidus level where feeding arteries terminate, resulting in the direct arteriovenous shunting seen surgically and pathologically. Additionally, the presence of arterialized draining veins is another key pathophysiologic marker. These arterialized veins appear fragile due to exposure to arterial blood and pressure and can be mistaken for arteries. Furthermore, both the arterial and venous components of AVMs express smooth muscle contractility markers like S2, increasing the risk of misidentifying veins as arteries (Xie et al., 2022).

Population studies have elucidated the epidemiology and distribution of AVMs. One of the earliest was a 27-year retrospective analysis in Olmsted County, Minnesota, revealing an incidence of approximately 0.82 per 100,000 person-years based on individuals with hemorrhagic presentation. AVMs can present at any age with a roughly equal gender distribution. While a congenital condition, they most commonly come to clinical attention in the second through fourth decades of life.

Recent years have seen breakthroughs in understanding the embryological origin of AVMs. Previously thought to arise from persistent arteriovenous connections, studies now implicate aberrant vascular patterning and cell signaling related to gene mutations involved in maintaining, repairing, and remodeling embryonic vascular structures. The earlier these mutations occur, the higher the likelihood of multiple AVMs forming.

While isolated AVMs comprise over 90% of cerebrovascular malformations, multiple AVMs often associate with genetic syndromes like hereditary hemorrhagic telangiectasia (HHT) or capillary malformation-arteriovenous malformation (CM-AVM). Given the patient’s prior history of pulmonary angiography and embolization, whole exome sequencing was performed, revealing the fetal multiple AVMs were likely related to the mother’s pathogenic ENG variant associated with HHT.

HHT has three subtypes: type 1 is caused by ENG mutations encoding endoglin, a TGF-beta co-receptor frequently involving pulmonary and neurologic AVMs; type 2 results from ACVRL1 mutations often with pulmonary hypertension and hepatic involvement; these two account for ∼85% of clinically suspected and genetically confirmed cases. Type 3 maps to chromosome 5q31 but the gene remains unknown (Arthur and Roman, 2022). An important consideration in this case is the presence of polydactyly and an accessory auricle in the fetus, features not typically associated with classic HHT. This raised the possibility of a more complex overlapping syndrome or a contiguous gene deletion. However, a thorough review of the WES data and chromosomal microarray analysis (CMA) did not identify other causative genetic alterations. Therefore, while a coincidental co-occurrence cannot be entirely ruled out, these findings may speculatively represent a rare expansion of the phenotypic spectrum associated with this specific ENG variant. Given the mother’s definitive clinical history of pulmonary AVMs and the identification of a known pathogenic ENG mutation in both mother and fetus, HHT remains the most compelling and primary diagnosis. This highlights the importance of comprehensive clinical and genetic evaluation when atypical features are present.

A significant finding of this report is the re-evaluation of the ENG variant c.1088G>A (p.Cys363Y). The diagnostic laboratory initially classified it as a Variant of Uncertain Significance (VUS). However, according to ACMG guidelines, evidence of co-segregation with disease in a family is a strong criterion for pathogenicity. In this case, the mother, a carrier of the variant, has a history of severe pulmonary AVMs requiring surgical intervention, while the fetus, who also inherited the variant, presented with multiple, severe cerebral AVMs. The combination of strong familial co-segregation and existing clinical reports provides compelling evidence to upgrade the classification of this variant from VUS to “Likely Pathogenic.” This case thereby contributes to a clearer understanding of the pathogenicity of this specific ENG variant.

The identification of a specific pathogenic ENG variant in this family naturally leads to a discussion of future etiological treatments, including gene therapy. While current management of HHT is primarily symptomatic, focusing on treating active bleeding and managing AVMs, the monogenic nature of the disease makes it a candidate for advanced therapeutic strategies. Promising preclinical research has explored gene therapy for HHT-associated AVMs. One innovative approach utilizes an adeno-associated viral vector (AAV9) to deliver soluble FLT1 (sFLT1), a protein that binds to and neutralizes vascular endothelial growth factor (VEGF), a key pro-angiogenic factor implicated in AVM formation. In mouse models where brain AVMs were induced through Eng gene deletion, this gene therapy significantly reduced the number of abnormal vessels, decreased the incidence of AVMs, and improved survival rates. This demonstrates the potential of targeting downstream effects of the genetic mutation. Other theoretical gene therapy avenues include direct correction of the mutant allele using technologies like CRISPR/Cas9, although this currently carries significant risks of off-target effects. For specific types of mutations, such as nonsense mutations that create a premature stop codon, therapies enabling stop-codon read-through are also being explored.

The confirmation of a pathogenic ENG variant as the cause of HHT in this family highlights the significant progress made since the discovery of the genetic basis of this disease. While current management of HHT is often symptomatic, focusing on embolization or surgical treatment of AVMs, research is increasingly focused on etiological therapies that target the underlying molecular pathology. Mutations in ENG or ACVRL1 disrupt the BMP9/10-ALK1-Smad1/5/9 signaling pathway, a critical component of the TGF-β superfamily that is essential for maintaining vascular quiescence. This disruption leads to a pro-angiogenic state, providing a clear rationale for mechanism-based treatments aimed at restoring pathway function or inhibiting downstream angiogenic drivers.

Several classes of drugs targeting this pathway are now in development or clinical use:Anti-angiogenic Agents: This is the most established category of etiological treatment. Intravenous Bevacizumab, a monoclonal antibody against the pro-angiogenic factor VEGF, is now widely used to manage severe bleeding and high-output cardiac failure in HHT patients. Furthermore, oral tyrosine kinase inhibitors like Pazopanib, which targets the VEGF receptor, and immunomodulatory drugs such as Pomalidomide, have shown promise in clinical trials for reducing bleeding severity by inhibiting pro-angiogenic signaling. BMP Pathway Modulators: More directly addressing the core genetic defect, some therapies aim to reactivate the deficient signaling cascade. Tacrolimus, an immunosuppressant, has been identified as a potent activator of the BMP9-ACVRL1-SMAD1/5/8 signaling pathway and can increase the expression of the normal ENG allele *in vitro*. By acting as an ALK1 signaling mimetic, it holds promise for restoring vascular stability, and clinical trials are currently evaluating its efficacy for HHT-related epistaxis. Novel Preclinical Targets: Preclinical research continues to identify new therapeutic targets. For instance, studies in HHT animal models have shown that pharmacological blockade of Angiopoietin-2 (Ang2), another key regulator of angiogenesis, can prevent and even reverse vascular lesions, suggesting it as a promising future therapeutic target.

Fetal AVMs can increase circulatory volume load, particularly on the right ventricle. They can also compress adjacent brain tissue or cause ischemic injury by “stealing” blood away from normal parenchyma through large shunting arteries. Hemolytic anemia and disseminated intravascular coagulation (Kasabach-Merritt phenomenon) are other potential complications.

Furthermore, counseling regarding the risk of recurrence is a critical component of management for this family. As HHT is an autosomal dominant disorder, there is a 50% risk of transmission to the fetus in each future pregnancy. Future prenatal strategies should be discussed, including the options of preimplantation genetic diagnosis (PGD) or prenatal diagnosis via chorionic villus sampling or amniocentesis to test for the familial ENG mutation. For any future pregnancy, regardless of genetic testing, we would recommend serial fetal monitoring with high-resolution ultrasound, including detailed neurosonography and fetal echocardiography, to assess for the development of AVMs and associated signs of high-output cardiac compromise.

In conclusion, this case of multiple AVMs is notable for its rarity and highlights the significant clinical challenges and high risk of adverse natural history and cardiac decompensation these lesions pose, underscoring the necessity of a multidisciplinary approach at tertiary fetal/neonatal brain anomaly referral centers. This is critical for accurate diagnosis to guide monitoring and obstetric management strategies based on the risks of adverse fetal outcome. Moreover, precise phenotyping of the vascular lesions and evaluation of other systemic manifestations in the proband and family, including bleeding history and careful skin examination for telangiectasias, is essential for appropriate genetic counseling which could profoundly impact other at-risk undiagnosed relatives through early diagnosis, treatment and more informed reproductive planning.

**Established facts and novel insights**

**Established facts**

•Already known fact 1. Arteriovenous malformations (AVMs) are rare but significant vascular anomalies that can occur in the brain, often leading to complications such as hemorrhage and ischemia.
•Already known fact 2. Most AVMs are sporadic and can occur in various parts of the brain, with a limited understanding of the genetic factors involved in familial cases.
•Already known fact 3. Hereditary hemorrhagic telangiectasia (HHT) is a genetic condition associated with multiple AVMs, particularly in the brain, lungs, and other organs. It is caused by mutations in genes like encoding endoglin (ENG), which are involved in vascular development and remodeling.

**Novel insights**

•New information 1. This case highlights the potential maternal genetic susceptibility to fetal cerebral AVMs, linking a pathogenic ENG gene variant in the mother to the development of multiple fetal AVMs.
•New information 2. The identification of the ENG mutation in the mother and its possible role in the fetal manifestation of multiple AVMs provides new insights into the genetic basis of AVMs in familial contexts.
•New information 3. The findings suggest that early genetic testing and careful monitoring of at-risk pregnancies can help predict and manage the risks of fetal brain malformations.

## Data Availability

The original contributions presented in the study are included in the article/supplementary material, further inquiries can be directed to the corresponding author.
